# Supplementary material for: Feature-based detection of automated language models: tackling GPT-2, GPT-3 and Grover
Source: PeerJ Comput Sci. 2021 Apr 6;7:e443. doi: 10.7717/peerj-cs.443 (PMC8049133; doi:10.7717/peerj-cs.443)
Supplement: Supplemental Information 2 [file peerj-cs-07-443-s002.pdf]

Explanations to the code submitted with *Feature-Based Detection of Automated Language-Models: Trying GPT-2, GPT-3 and Grover*

The code accompanying the submission covers all steps necessary to replicate the results discussed in the submission, from downloading the different language model datasets and pre-processing, filtering and grouping them, over extracting the features and running validation trials to decide on the final model architecture, to training and evaluating the feature-based detection models. This document tries to serve as a handbook to the code, explaining its structure, the input and output of each stage and providing further information where necessary.

Downloading the data

The data used in this submission consists of language-model samples, generated and published by the authors and creators of the corresponding language models. At time of submission, the GPT-2 samples were hosted at https://storage.googleapis.com/gpt-2/output-dataset/v1/, the GPT-3 samples at https://raw.githubusercontent.com/openai/gpt-3/master/175b_samples.jsonl and the Grover samples at https://storage.googleapis.com/grover-models/generation_examples/generator=mega~dataset=p0.94.jsonl.
These addresses would need to be updated in the code should they ever change. In any case, private copies of the datasets would be available from the authors.
*Please note: To download the GPT-2 samples, it is necessary to concatenate the exact location of the desired file to the base URL provided above. The exact location is provided by replacing the placeholders in '**size**.**split**.jsonl' with a **size** from [webtext,small-117M,small-117M-k40,xl-1542M,xl-1542M-k40] and a **split** from [train,valid,test]. The test samples for the webtext data would thus be available from https://storage.googleapis.com/gpt-2/output-dataset/v1/webtext.test.jsonl. The download should commence automatically when navigation to this URL. The supplementary code does this automatically.*

To download the data and store it in the necessary file structure for running the subsequent code, the file *download.py* needs to be executed. If no further arguments are entered, the code defaults to downloading the individual datasets and creates from them the multi-dataset and filtered versions that are used in the corresponding experiments. d*ownload.py* otherwise takes 2 arguments, which can be used to control the described additional data preparation steps. The first argument takes True or False to control the generation of the multi-datasets, and the second argument takes True or False to control the generation of the filtered-versions.

Extracting the feature

Once the data is downloaded, the next step consists in extracting the features from it. Because of the way they are extracted and later used, both the *empath*- and *Q-features* need a pre-processing step. For the *Q*-features, an additional difficulty is their definition as corpus-based features. For any text, they thus always depend not only on the text itself, but also on the corpus the text is located within.

Therefore, *extract_empath.py*, *extract_Q_raw.py* and *extract_Q_features.py* should be executed.

extract_empath.py takes three mandatory arguments: *model*, *size*, *split*, and proceeds to create the empath pre-features accordingly. See the following table for allowed combinations.

Model	Size	Split
GPT2	*small-117M, small-117M-k40, xl-1542M, xl-1542M-k40, webtext*	*train, valid, test*
GPT3	*175B, GPT3_webtext*	*train, valid, test*
Grover	*groverMega, realNews, realNews_solo*	*train, valid, test*

extract_Q_raw.py can be used to either extract the corpus-based Q-features for single- or multi-source-datasets. If the first argument entered is *single*, specification of the *model* and *size* is needed as second and third arguments. The last mandatory argument is either *True* or *False* for a filter-dummy, extracting the Q-features either for the filtered- or non-filtered-corpus. If the first argument entered is *multi*, then the required arguments are *combi* and *source* to determine which corpus to extract the Q-features from. See the following table for allowed combinations.

Combi	Source
superGPT2, superGPT_un, superGPT_k, superAll	*machine, human*

After *extract_Q_raw.py* has been executed, *extract_Q_features.py* needs to be evoked to calculate the actual Q-feature-values for every text from the raw, corpus-based counts extracted in the previous step. e*xtract_Q_features.py* takes either *single* or *multi* as the first argument, depending on what corpus the Q-features should be extracted for. If the first argument is *single*, then 6 more arguments need to be given: *model, size, human_text, machine_text, split, filtered*. *model*, *size*, *split* and *filtered* determine for which dataset the Q-features should be extracted. *human_text* and *machine_text* always depend on each other, and are necessary to specify which corpus is responsible for the samples of human and which is for the samples of machine text. Either *human_text* or *machine_text* must thus be equivalent to the entered *size* argument. *model == 'GPT2'*, size == *'small-117M', human_text == 'webtext', machine_text == 'small-117M'* would therefore extract the Q-features for the *small-117M GPT-2* dataset, using the *small-117M* texts as machine-samples and webtext as human-samples. The allowed pairs of human- and machine-text are summarised in the following table. If the first argument is *multi*, then 3 more arguments need to be given: *combi*, *source* and *split*. The same *combi* and *source* combinations as stated above are allowed, and *split* must be one out of *train, valid* and *test*.

human_text	machine_text
webtext	*small-117M, small-117M-k40, xl-1542M, xl-1542M-k40*
GPT3_webtext	*175B*
realNews_solo	*groverMega*

Finally, after all previously mentioned pre-extraction steps have been executed successfully, the final feature vectors can be created by executing *extract_features.py*. The necessary arguments are *model, size* and *split*, determining the dataset for which the features should be extracted. Note that no (corpus-based) Q-features are to be extracted here, for they have been extracted previously and are concatenated to the main features extracted in this step as needed. All features are now extracted.

Running the validation trials

Validation trials have been done in order to determine which model architecture to use for the experiments conducted in the submission. Running *validation.py* recreates these validation trials, based on the previously extracted features of the four GPT-2 datasets.

Running the experiments

Single datasets classifiers

The main part of our experiments is based on the evaluation and comparison of the feature-based detector's performance on individual datasets. These results can be replicated using the code in *train_single.py*. The needed arguments are *model*, *human_text*, *machine_text*, *filtered* and *Q*. The first three arguments determine which machine-generated samples are detected against which samples of human text. Note that only the combinations introduced in the section on the extraction of Q-features are allowed. *filtered* and *Q* are True-False dummies to control whether the filtered versions of the datasets shall be used, and whether the corpus-based Q-features are to be included among the features used to train and test the classifiers.

Multi datasets classifiers

The section of our experiments that tests the performance of the feature-based classifier on datasets consisting of generations from different language models can be recreated by executing *train_multi.py*. The two mandatory arguments are *combi* and *Q*. *Combi* determines which mixture of individual datasets is to be considered, valid arguments are the same as listed in the table above. *Q* takes True or False as argument and controls whether Q-features are to be included in the features or not.

Feature-Set classifiers

The section of our experiments that tests the performance of the individual feature-subsets can be recreated by executing *train_featureSets.py*. The three mandatory arguments *model, human_text* and *machine_text* determine which of the available, individual datasets is to be used for training and testing the feature-set classifiers. The allowed combinations are as described above.

tfidf-baselines

To recreate the tf-idf baseline results, execute *train_tfidf.py*. The required arguments are *model, size, n* and *save. model* and *size* again determine which dataset to run the experiments for. *n* is used to set the size of the tf-idf vector that is to be used, and depends on the available memory. In the submission, *n* has been set to 100,000.

Ensemble classifiers

To recreate the ensemble-results, execture *train_ensemble.py*. The required arguments are *model*, *human_text*, *machine_text*, *n*, *architecture* and *ensemble*. As introduced before, *model, human_text* and *machine_text* determine which dataset to train and test an ensemble classifier on. *n* sets the size of the tf-idf vector used for the corresponding classifier and has been set to 100,000 during the submission's experiments with the GPT-2 data, due to memory constraints. For GPT-3 and Grover it should be set to the largest possible *n*, 1604 and 8000 respectively. *architecture* can be set to either *NN* or *LR* and controls the architecture used for the ensemble classification model. e*nsemble* can be set to either *super* or *separate* and determines the structure of the ensemble classification model, as described in the submission.
