# Supplementary material for: Feature-based detection of automated language models: tackling GPT-2, GPT-3 and Grover
Source: PeerJ Comput Sci. 2021 Apr 6;7:e443. doi: 10.7717/peerj-cs.443 (PMC8049133; doi:10.7717/peerj-cs.443)
Supplement: Supplemental Information 3 [file peerj-cs-07-443-s003.pdf]

Features

This document provides additional examples and explanations for selected features as well as a comprehensive overview of all features that have been used for classification, categorized by the different feature-sets and referenced to the type of artifact produced by language models they are expected to exploit.

Conjunction Overlap captures repetitions of Uni-, Bi- and Trigrams around *and*-conjunctions; *the text and the text*

Coreference Chains (or clusters) are based on the repeated reference towards an enitity throughout the text. References can be made in a number of ways, the most common include the use of the entity's name, a descriptive noun (phrase) or pronouns. The span of a coreference chain is the difference in index-position of the first and last reference towards the coreference chain's entity in a text; *Emma writes a book. She aspires to become a famous author.* In this example, a coreference chain concerned with the entity referred to as Emma can be found. The two references made to that entity are unique, and the first is a *named entity* reference. The span of this coreference chain is 4, as the first reference sits on index-position 0 and the second reference sits on index-position 4.

Empath Features are simple, word-count based features. Given a number of words defining a category, e.g. *blue - green - red - yellow* for the category *colors*, the number of occurences of words from that category in a text are summed up to yield that category's empath score; *The red ball was thrown into the blue sea* would thus have an empath score of 2 in the *colors*-category.

Entity-Grid Features build on the relative frequencies of entity transitions through a text. In addition to just tracking their appearance, entity transitions also consider the entities' grammatical role; *Tom likes to tell jokes. He is considered to be funny.* Since the entity referred to as Tom appears as a subject in the first sentence and reappears as an object in the consecutive sentence, an entity transition from subject [S] to object [O] would be registered here.

Index	Feature
basic features (absolute) (Other Features)	
0	Number of characters
1	Number of syllables
2	Number of words
3	Number of sentences
4	Number of difficult words
5	Number of short words
6	Number of long words
basic features (relative) (Other Features)	
7	Characters per Word
8	Syllables per Word
9	Words per Sentence
10	Share difficult words in total words
11	Share short words in total words
12	Share long words in total words
readability features (Other Features)	
13	Automatic Readability Index
14	Coleman Liau Index
15	Flesch-Kincaid Grade Level
16	Flesch-Kincaid Reading Ease
17	Gunning-Fog Index
18	LIX
19	McAlpine EFLAW Score
20	RIX
21	SMOG Grade
lexical diversity features (Repetitiveness)	
22	Share stop-words in total words
23	Share unique words in total words
24	Share words in google top-100 list in total words
25	Share words in google top-1000 list in total words
26	Share words in google top-10000 list in total words
formatting features (Other Features)	
27 - 39	Rel. frequencies of punctuation marks [,.:;?!-”()[]\n]
40 - 52	Punctuation marks per sentence
53	Number of paragraphs
54	Average paragraph length

Table 1: Feature Overview I

Index	Feature
lexical and syntactic repetitiveness features (Repetitiveness)	
55 - 64	Unigram overlap of words between consecutive sentences (10 uniform bins from 0 to 1)
65 - 74	Bigram overlap of words between consecutive sentences (10 uniform bins from 0 to 1)
75 - 84	Trigram overlap of words between consecutive sentences (10 uniform bins from 0 to 1)
85 - 94	Unigram overlap of POS-tags between consecutive sentences (10 uniform bins from 0 to 1)
95 - 104	Bigram overlap of POS-tags between consecutive sentences (10 uniform bins from 0 to 1)
105 - 114	Trigram overlap of POS-tags between consecutive sentences (10 uniform bins from 0 to 1)
115 - 117	Uni-, Bi- and Trigram overlap of words around *and*-conjunctions
syntactic features (Lack of Syntactic and Lexical Diversity)	
118 - 136	Rel. frequencies of POS-tags [ADJ, ADP, ADV, NOUN, VERB, AUX, CONJ, CCONJ, DET, INTJ, NUM, PART, PRON, PROPN, PUNCT, SCONJ, SYM, X, SPACE]
137 - 155	POS-tags per sentence
156 - 160	[ADJ,ADP,ADV,NOUN,VERB]-tags in total words
161 - 165	Unique [ADJ,ADP,ADV,NOUN,VERB]-tags in total words
166 - 170	[ADJ,ADP,ADV,NOUN,VERB]-tags in total [ADJ,ADP,ADV,NOUN,VERB]-tags
171 - 175	Unique [ADJ,ADP,ADV,NOUN,VERB]-tags in total unique [ADJ,ADP,ADV,NOUN,VERB]-tags
176 - 180	[ADJ,ADP,ADV,NOUN,VERB]-tags per sentence
181 - 185	Unique [ADJ,ADP,ADV,NOUN,VERB]-tags per sentence
named-entity features (Lack of Syntactic and Lexical Diversity)	
186 - 203	Rel. frequencies of NE-tags [PERSON, NORP, FAC, ORG, GPE, LOC, PRODUCT, EVENT, WORK-OF-ART, LAW, LANGUAGE, DATE, TIME, PERCENT, MONEY, QUANTITY, ORDINAL, CARDINAL]
204 - 221	NE-tags per sentence
222	Share unique NE-tags in total NE-tags
223	NE-tags in total words
224	Unique NE-tags in total words
225	NE-tags in total sentences
226	Unique NE-tags in total sentences
coreference features (Lack of Syntactic and Lexical Diversity)	
227 - 236	Share of unique coreferences in total coreferences per cluster (10 uniform bins from 0 to 1)
237	Coreferences per cluster
238	Average span of clusters
239	Share of long coreference chains (span > document length / 2)
240	Share of short inferences (distance between first and second coreference ≤ 20)
241	Share of shorter inferences (distance between first and second coreference ≤ 10)
242	Share of shortest inferences (distance between first and second coreference ≤ 5)
243	Share of NEs in total references
244	Active coreference chains per word
245	Active coreference chains per NE-tag

Table 2: Feature Overview II

Index	Feature
entity-grid features (Lack of Coherence)	
246 - 261	Rel. frequencies of entity transitions [SS, SO, SX, S-, OS, OO, OX, O-, XS, XS, XX, X-, -S, -X, -O, –]
topic redundancy features (Lack of Coherence)	
262	Information Loss
263 - 266	Mean, Median, Maximum and Minimum of truncated Matrix
267 - 270	Difference in Mean, Median, Maximum and Minimum between original and truncated Matrix
271	Information Loss (lemmatized)
272 - 275	Mean, Median, Maximum and Minimum of truncated Matrix (lemmatized)
276 - 279	Difference in Mean, Median, Maximum and Minimum between original and truncated Matrix (lemmatized)
empath features (Lack of Purpose)	
280	Share of topical words in total words
281 - 285	Mean, Median, Minimum, Maximum and Variance of empath scores
286	Number of active categories (empath score > 0)
287 - 291	Mean, Median, Minimum, Maximum and Variance of active categories
292 - 296	Empath scores of [spatial,sentiment,opinion,logic,ethic] categories
yule's Q features (Lack of Coherence)	
297	Q-Score based on human corpus
298	Q-Score based on machine corpus
299	Share of word-pairs not in human corpus
300	Share of word-pairs not in machine corpus

Table 3: Feature Overview III
