# Supplementary material for: Feature-based detection of automated language models: tackling GPT-2, GPT-3 and Grover
Source: PeerJ Comput Sci. 2021 Apr 6;7:e443. doi: 10.7717/peerj-cs.443 (PMC8049133; doi:10.7717/peerj-cs.443)
Supplement: Supplemental Information 4 [file peerj-cs-07-443-s004.pdf]

Optimization

The first table documents the parameter grid underlying the grid-search to find the best individually optimized classifier across a range of parameter constellations. The resulting optimal parameter constellations for the different classifiers are documented in the subsequent tables.

Parameter	Values
Layers	(100), (25,50,25)
Activation	ReLU, Logistic
Learning Rate	0.001, 0.01
Alpha	0.00005, 0.0001, 0.0005

Table 1: Grid-Search Parameters.

Classifier	Parameters			
	Layers	Activation	Learning Rate	Alpha
s	(25, 50, 25)	ReLU	0.01	0.0005
xl	(100)	ReLU	0.01	0.0005
s-k	(100)	ReLU	0.001	0.0005
xl-k	(25, 50, 25)	ReLU	0.01	0.0005
GPT3	(100)	Logistic	0.001	0.0005
Grover	(25, 50, 25)	ReLU	0.01	0.0001

Table 2: Single-Dataset Classifiers, Optimal Parameter Constellations.

Classifier	Parameters			
	Layers	Activation	Learning Rate	Alpha
s	(100)	Logistic	0.001	0.0005
xl	(25, 50, 25)	ReLU	0.001	0.00005
s-k	(25, 50, 25)	ReLU	0.001	0.0005
xl-k	(25, 50, 25)	ReLU	0.001	0.00005
GPT3	(25, 50, 25)	Logistic	0.001	0.0001
Grover	(100)	ReLU	0.01	0.00005

Table 3: Single-Dataset Classifiers, no Q, Optimal Parameter Constellations.

Classifier	Parameters Layers	Activation	Learning Rate	Alpha
s	(25, 50, 25)	ReLU	0.001	0.0005
xl	(100)	ReLU	0.01	0.0005
s-k	(100)	ReLU	0.001	0.0005
xl-k	(100)	ReLU	0.01	0.00005
GPT3	(100)	ReLU	0.01	0.00005
Grover	(100)	Logistic	0.091	0.0001

Table 4: Single-Dataset Classifiers, Filtered, Optimal Parameter Constellations.

Classifier	Parameters Layers	Activation	Learning Rate	Alpha
GPT2-un	(25, 50, 25)	ReLU	0.01	0.0005
GPT2-k	(100)	ReLU	0.01	0.0005
GPT2	(25, 50, 25)	ReLU	0.01	0.0005
All	(100)	ReLU	0.01	0.0001

Table 5: Multi-Dataset Classifiers, Optimal Parameter Constellations.

Classifier	Parameters Layers	Activation	Learning Rate	Alpha
GPT2-un	(25, 50, 25)	ReLU	0.001	0.0001
GPT-k	(25, 50, 25)	ReLU	0.001	0.0001
GPT2	(25, 50, 25)	ReLU	0.001	0.00005
All	(25, 50, 25)	ReLU	0.001	0.00005

Table 6: Multi-Dataset Classifiers, no Q, Optimal Parameter Constellations.

Feature-Set	Classifier s	xl	s-k	xl-k	GPT3	Grover
basicAbs	0.001	0.001	0.001	0.001	0.001	0.001
	0.0001	0.00005	0.0001	0.00005	0.0001	0.0001
basicRel	0.001	0.0001	0.001	0.001	0.001	0.001
	0.00005	0.0001	0.0001	0.00005	0.00005	0.0001
readability	0.001	0.001	0.001	0.001	0.001	0.0001
	0.0001	0.00005	0.00005	0.00005	0.0001	0.0001
lexicalDiv	0.001	0.001	0.001	0.001	0.0001	0.001
	0.00005	0.0001	0.00005	0.00005	0.00005	0.0001
formatting	0.001	0.001	0.001	0.001	0.001	0.001
	0.00005	0.00005	0.00005	0.0001	0.00005	0.00005
repetitiveness	0.001	0.001	0.001	0.001	0.0001	0.001
	0.00005	0.0001	0.00005	0.00005	0.00005	0.00005
syntactic	0.001	0.001	0.001	0.001	0.001	0.001
	0.0001	0.00005	0.00005	0.00005	0.00005	0.0001
NE	0.001	0.001	0.001	0.0001	0.001	0.001
	0.0001	0.0001	0.0001	0.00005	0.0001	0.00005
coreference	0.001	0.001	0.001	0.0001	0.001	0.001
	0.00005	0.0001	0.0001	0.00005	0.00005	0.0001
entityGrid	0.001	0.001	0.001	0.0001	0.0001	0.001
	0.00005	0.0001	0.0001	0.0001	0.00005	0.00005
infoLoss	0.001	0.001	0.001	0.001	0.001	0.001
	0.0001	0.00005	0.0001	0.00005	0.00005	0.00005
empath	0.001	0.001	0.001	0.0001	0.001	0.001
	0.0001	0.0001	0.00005	0.00005	0.0001	0.0001
Q	0.0001	0.001	0.0001	0.0001	0.001	0.0001
	0.00005	0.00005	0.00005	0.00005	0.00005	0.00005

Table 7: Feature-Set Classifiers, Optimal Parameter Constellations. The feature-set classifiers have been optimised only on the initial learning rate (Values: [0.0001, 0.001]) and the alpha parameter (Values: [0.00005, 0.00001]), with the activation being fixed to *ReLU* and the layers to *(25,50,25)*. For every set of features, the first row shows the optimal initial learning rate, and the second row the optimal alpha parameter.

Classifier	LR C	NN Activation	Layers	Learning Rate	Alpha
**Separate**					
s	1/64	Logistic	(5, 10, 5)	0.00001	0.00001
xl	1/64	Logistic	(5, 10, 5)	0.00001	0.00001
s-k	32	Logistic	(5, 10, 5)	0.0001	0.00001
xl-k	64	ReLU	(100)	0.00001	0.00001
GPT3	1	Logistic	(25, 50, 25)	0.001	0.00001
Grover	64	Logistic	(100)	0.001	0.00001
**Super**					
s	1/64	ReLU	(100)	0.0001	0.00001
xl	1/8	Logistic	(25, 50, 25)	0.001	0.00001
s-k	4	ReLU	(5, 10, 5)	0.0001	0.005
xl-k	1/64	Logistic	(100)	0.001	0.005
GPT3	1/8	Logistic	(25, 50, 25)	0.001	0.00001
Grover	0.25	Logistic	(5, 10, 5)	0.001	0.00001

Table 8: Ensemble-Classifier, Optimal Parameter Constellations. The NN ensemble-classifiers have been optimised on the type of activation (Values: [ReLU, Logistic]), the hidden layer sizes (Values: [(100), (25,50,25), (5, 10, 5)]), the initial learning rate (Values: [0.00001, 0.0001, 0.001] and alpha (Values: [0.00001, 0.00005, 0.0001, 0.0005]). The LR ensemble-classifiers have been optimised on the regulation parameter C. Values: [1/64, 1/32, 1/16, 1/8, 1/4, 1/2, 1, 2, 4, 8, 16, 32, 64]
